# Coronary angiography and reperfusion therapy following out-of-hospital cardiac arrest in women

**DOI:** 10.1016/j.resplu.2026.101462

**Published:** 2026-08-24

**Authors:** Charlotte Miedel, Lis Abazi, Sune Forsberg, Martin Jonsson, Gabriel Riva

**Affiliations:** a Department of Clinical Science and Education, Karolinska Institutet, Södersjukhuset Sjukhusbacken 10, 118 83 Stockholm, Sweden; b Department of Anesthesiology and Intensive Care, Norrtälje Hospital Lasarettsgatan, 761 45 Norrtälje, Sweden; c Department of Cardiology, Capio Saint Göran’s Hospital Sankt Göransplan 1, 112 19 Stockholm, Sweden

**Keywords:** Out-of-hospital cardiac arrest, Post-resuscitation care, Coronary angiography, Reperfusion therapy, Sex differences, Registry-based study

## Abstract

**Introduction:**

Survival after out-of-hospital cardiac arrest is lower in women than in men, often attributed to older age, more comorbidities and non-shockable rhythms at presentation. Coronary angiography and reperfusion therapy are cornerstones in post-cardiac arrest care yet appear to be used less often in women. Data on coronary artery disease among patients presenting with non-shockable rhythms and no ST-segment elevation remain limited.

**Methods:**

We conducted a nationwide cohort study including all out-of-hospital cardiac arrest patients admitted alive to Swedish hospitals between 2010 and 2019. Adjusted odds ratios (OR) of coronary angiography and percutaneous coronary intervention during hospitalization were calculated for women compared with men, controlling for age, comorbidities, and initial rhythm. Sex-specific angiographic findings were examined in patients without shockable rhythm or ST-segment elevation.

**Results:**

Of 8641 patients, 29.4% were women. Coronary angiography was performed in 31.5% of women versus 51.0% of men. Women were less likely to undergo coronary angiography (OR 0.77, 95% CI 0.66–0.90) and percutaneous coronary intervention (OR 0.76, 95% CI 0.64–0.90). After adjusting for angiographic findings, intervention rates were similar between sexes (OR 0.97, 95% CI 0.80–1.18). Among angiographed patients with non-shockable rhythms and no ST-elevation, significant coronary artery disease was found in 31% of women and 53% of men.

**Conclusion:**

Women with resuscitated out-of-hospital cardiac arrest were less likely to undergo coronary angiography yet had similar rates of percutaneous coronary intervention when obstructive coronary artery disease was present. In patients without an initial shockable rhythm or ST-segment elevation, many had significant coronary artery disease.

## Background

Out-of-hospital cardiac arrest (OHCA) is associated with very high mortality, with only around 10% of patients surviving to hospital discharge.^1^ Among women, OHCA prognosis is even worse, with survival rates around 6% compared to 11% among men.^2^ The sex difference in survival might in part be explained by the fact that women are older at the time of the cardiac arrest, and less likely to present with shockable initial rhythm, i.e. ventricular tachycardia (VT) or ventricular fibrillation (VF).^3^, ^4^

Acute myocardial infarction is a common underlying cause of OHCA, and coronary artery disease (CAD) is highly prevalent among OHCA survivors, particularly in patients presenting with ST-segment elevation on the first registered electrocardiogram (ECG) and/or shockable rhythms.^5^, ^6^ In these patients, early coronary angiography and revascularization is associated with improved outcome and supported by current guidelines.^7^, ^8^, ^9^ Studies have shown that female OHCA victims with return of spontaneous circulation (ROSC) are less likely to undergo coronary angiography and reperfusion therapy compared to men, however data are conflicting.^3^, ^10^, ^11^, ^12^

OHCA victims presenting with non-shockable first rhythms (pulseless electrical activity or asystole) and no ST-segment elevation on the first registered ECG represent a large and challenging group of patients. Despite having the worst prognosis, they receive the least invasive cardiac care.^5^, ^13^ Randomized trials have not shown a benefit of the performance of routine early coronary angiography in patients without ST-segment elevation, however patients with non-shockable rhythms have been underrepresented in several of these studies, and limited data suggest that significant CAD may still be present in a substantial proportion.^6^, ^7^, ^14^ Among the few who undergo coronary angiography in this subgroup, a culprit lesion requiring PCI have been reported in up to 1 out of 4 patients.^15^ This OHCA phenotype is particularly common among women, nevertheless, no previous study has provided sex-disaggregated data on coronary angiography rates, PCI or CAD prevalence in this subgroup of patients.

### Aim

The objective of this study was to assess sex differences in the utilization of coronary angiography and reperfusion therapy after OHCA in a nationwide cohort study in Sweden. Second, we aimed to describe coronary angiographic indications and findings in women and men presenting without ST-segment elevation and without a shockable initial rhythm.

## Methods

### Design

This was a registry-based cohort study including all patients with OHCA who were treated by emergency medical services (EMS) and admitted alive to hospital in Sweden between January 1, 2010, and December 31, 2019. Exclusion criteria were age <18 years, missing data on personal identification number, and missing data on sex. The study population was retrieved from the Swedish Registry for Cardiopulmonary Resuscitation (SRCR), which collects data on EMS-treated OHCA patients where cardiopulmonary resuscitation (CPR) and/or defibrillation is performed. All EMS services in Sweden report to SRCR through a web-based form or through direct data from medical charts. Immediate outcomes such as death or admission alive to hospital are reported by the EMS crew, where the latter was used as a surrogate measure for a successful resuscitation, i.e. ROSC. The registry has been described in detail elsewhere.^16^ SRCR was linked with other national healthcare registries using the Swedish personal identification number, which is unique to each individual in Sweden, to gather data on exposure, comorbidities, and outcomes.

### Exposure and outcome

The exposure was female sex, and the comparison group was male sex. The primary outcomes were performed coronary angiography and reperfusion therapy, i.e. PCI or CABG respectively, during the hospital course, as recorded in the Swedish Coronary Angiography and Angioplasty registry (SCAAR) for angiography and PCI, and as recorded in SRCR for CABG. Coronary angiography and PCI performed within 7 days after cardiac arrest was included in the analysis. Details on coronary angiography indications, findings, and decisions were described in women and men, respectively, using variables retrieved from SCAAR. A stratified descriptive analysis was conducted in patients with non-shockable rhythm and no ST-segment elevation to describe coronary angiographic indications, findings and decisions. SCAAR collects data from all centers performing coronary angiography and PCIs in Sweden. The register covers 100% of patients undergoing coronary angiography and angioplasty for any clinical indication and is routinely validated against patient records. It includes approximately 150 variables. Data on ECG findings were collected from SCAAR and is usually reported by the catheterization lab team, prior, during or after coronary angiography. The presence or absence of STEMI was defined based on the database reported reason for catheterization. The registry has been described in detail elsewhere.^17^

### Confounders

Potential confounders were age, calendar year (both continuous) and presenting rhythm (VT/VF, coded as yes or no), as recorded in the SRCR. Moreover, hypertension, diabetes mellitus, ischemic heart disease, chronic kidney disease, congestive heart failure, and chronic obstructive pulmonary disease were used as confounders (all coded as yes or no). These variables were retrieved from a 10-year historical search in the Swedish National Patient Registry using ICD-codes (as specified in Supplementary Table 2) or, if present, in SCAAR. The Swedish National Patient Registry (NPR) systematically collects data on diagnoses, procedures, and patient visits to specialized outpatient clinics and in-hospital admissions nationwide. It has been thoroughly validated, showing high diagnostic accuracy and minimal missing data.^18^, ^19^

### Statistical analyses

Logistic regression was used to estimate odds ratios (OR) and 95% confidence intervals (CI) for the outcome. One crude model and two adjusted models were used: one adjusted for age, calendar year, and comorbidities (specified under “Confounders”) and one adjusted for age, calendar year, comorbidities, and presenting rhythm. Statistical analyses were performed using R studio version 4.4.0 (2024.04.1+748).

### Ethics

Ethical approval for the study was granted by the Swedish ethical review authority (dnr 2022-02905-0).

## Results

### Patients

**Fig. 1:**
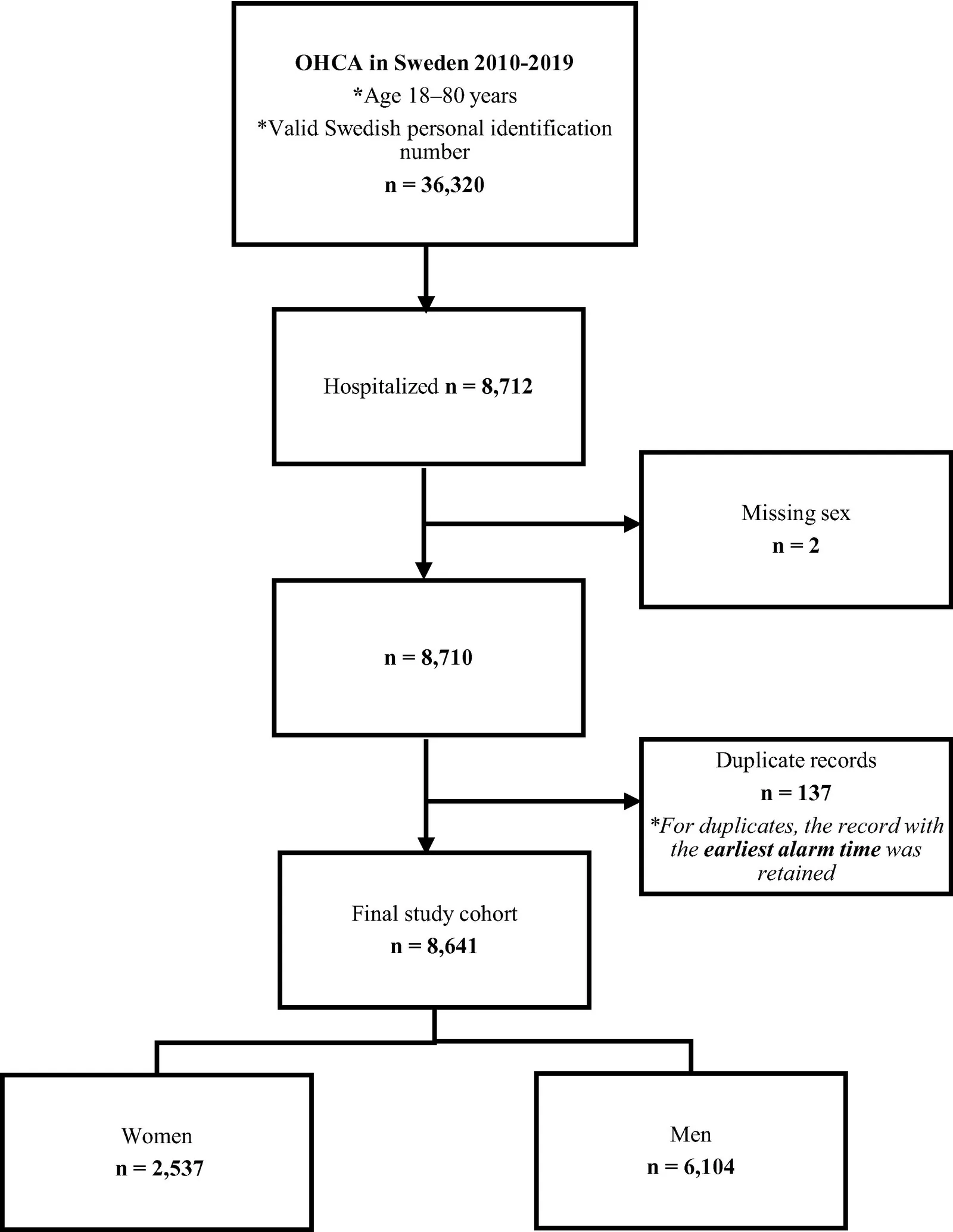
**Flowchart of patient inclusion and exclusion**. *Abbreviations:* OHCA, out-of-hospital cardiac arrest.

In total, 8641 EMS-treated OHCA patients who were admitted alive to hospital were included in the study (Fig. 1). Among these, 2537 (29.4%) were women. Baseline patient and resuscitation characteristics are presented in Table 1. Notable differences between women and men were seen in the distribution of hypertension, ischemic heart disease, heart failure, and chronic obstructive pulmonary disease, as well as in shockable initial rhythm and location of the cardiac arrest. Fewer women were discharged alive compared to men (37.4% versus 53.4%), while 1-day survival rates were similar between women and men (86.1% versus 89.8%; data not shown).

**Table 1:** Characteristics among 8641 resuscitated out-of-hospital cardiac arrest patients.

**Characteristic**	**Women**	**Men**
Total	2537 (29.4%)	6104 (70.6%)
Age, median [IQR]	66 [54–73]	65 [54–72]
Hypertension	455 (17.9%)	1680 (27.5%)
Diabetes mellitus	148 (5.8%)	400 (6.6%)
Ischemic heart disease	405 (16.0%)	1735 (28.4%)
Heart failure	324 (12.8%)	936 (15.3%)
Chronic kidney disease	103 (4.1%)	262 (4.3%)
Chronic obstructive pulmonary disease	371 (14.6%)	390 (6.4%)
Bystander CPR	1328 (52.3%)	3561 (58.3%)
Shockable initial rhythm	788 (31.1%)	3212 (52.6%)
Witnessed arrest	1958 (77.2%)	4983 (81.6%)

**Location of the arrest**		
At home	1694 (66.8%)	3177 (52.0%)
Public	401 (15.8%)	1908 (31.3%)
Other	441 (17.4%)	1014 (16.6%)

**Cardiac arrestaetiology (as reported)**		
Medical	2013 (79.3%)	5122 (83.9%)
Trauma	42 (1.7%)	116 (1.9%)
Intoxication	140 (5.5%)	285 (4.7%)
Drowning	35 (1.4%)	71 (1.2%)
Asphyxia	151 (6.0%)	179 (2.9%)
Other	60 (2.4%)	106 (1.7%)

**Outcomes**		
Coronary angiography	800 (31.5%)	3114 (51.0%)
Percutaneous coronary intervention (% of patients undergoing coronary angiography)	441 (55.1%)	2003 (64.3%)
CABG, performed or planned	31 (1.2%)	197 (3.2%)
Survival to hospital discharge	949 (37.4%)	3257 (53.4%)

*Abbreviations:* IQR, interquartile range; CPR, cardiopulmonary resuscitation; CABG, coronary artery bypass grafting.

### Coronary angiography findings and reperfusion therapy in women and men

Overall coronary angiography was performed in 800 (31.5%) women and 3114 (51.0%) men. Most coronary angiographies were performed on the same day as the cardiac arrest (78.3%). Characteristics among women and men undergoing coronary angiography are presented in Table 2. Among those undergoing coronary angiography, notable sex differences were seen in the distribution of ischemic heart disease and chronic obstructive pulmonary disease (Table 2). Moreover, women undergoing coronary angiography were less likely to present with shockable initial rhythm (67.9% versus 77.1%), less likely to receive bystander CPR (52.6% versus 61.0%), and less likely to suffer the cardiac arrest at a public location compared to men (21.1% versus 36.6%).

**Table 2:** Characteristics among 3914 resuscitated out-of-hospital cardiac arrest patients undergoing coronary angiography.

**Characteristic**	**Women**	**Men**
Total	800 (20.4%)	3114 (79.6%)
Median age, years [IQR]	65 [56–71]	65 [57–71]
Hypertension	346 (43.3%)	1320 (42.4%)
Diabetes mellitus	117 (14.6%)	511 (16.4%)
Ischemic heart disease	204 (25.5%)	1118 (35.9%)
Heart failure	73 (9.1%)	324 (10.4%)
Chronic kidney disease	14 (1.8%)	75 (2.4%)
Chronic obstructive pulmonary disease	55 (6.9%)	100 (3.2%)

**Smoking status**		
Never smoked	184 (23.0%)	720 (23.1%)
Ex smoker >1 month	105 (13.1%)	577 (18.5%)
Current smoker	180 (22.5%)	521 (16.7%)
Unknown	331 (41.4%)	1296 (41.6%)
Previous PCI	60 (7.5%)	397 (12.7%)
Previous CABG	26 (3.3%)	253 (8.1%)

**Resuscitation characteristics**		
Witnessed arrest	702 (87.8%)	2813 (90.3%)
Bystander CPR	421 (52.6%)	1898 (61.0%)

**Location of the arrest**		
At home	451 (56.4%)	1343 (43.1%)
Public	169 (21.1%)	1139 (36.6%)
Other	180 (22.5%)	629 (20.2%)
Shockable initial rhythm	543 (67.9%)	2401 (77.1%)

**Cardiac arrestaetiology (as reported)**		
Medical	757 (94.6%)	2956 (94.9%)
Trauma	3 (0.4%)	18 (0.6%)
Intoxication	5 (0.6%)	11 (0.4%)
Drowning	0 (0.0%)	4 (0.1%)
Asphyxia	4 (0.5%)	12 (0.4%)
Other	2 (0.3%)	2 (0.1%)

**Survival**		
Survival to hospital discharge	520 (65.0%)	2256 (72.4%)

*Abbreviations:* IQR, interquartile range; CPR, cardiopulmonary resuscitation; PCI, percutaneous coronary intervention; CABG, coronary artery bypass grafting.

The reported indications for coronary angiography were similar between sexes (Table 3). Women who underwent coronary angiography had more normal coronary angiograms compared to men, while men had more 2, 3, or advanced vessel disease (Table 3). Of those undergoing coronary angiography, 57% women underwent reperfusion therapy (i.e. PCI ad hoc, elective PCI or CABG), as compared to 69% among men (Table 3). Survival to hospital discharge was higher among women and men undergoing coronary angiography compared to the entire cohort, although the proportion of women surviving to hospital discharge was still lower than that of men (65.0% versus 72.4%, unadjusted) (Table 1, Table 2).

**Table 3:** Coronary angiography indications, findings and primary decision.

	**Women**	**Men**
Total	800	3114

**Indication**		
ST-elevation myocardial infarction	194 (24.3%)	733 (23.5%)
Cardiac arrest w STEMI	166 (20.8%)	657 (21.1%)
Cardiac arrest w/o STEMI	246 (30.8%)	950 (30.5%)
Cardiac arrest, undefined	114 (14.3%)	483 (15.5%)
NSTEMI/unstable angina pectoris	25 (3.1%)	93 (3.0%)
Due to arrhythmia	28 (3.5%)	111 (3.6%)
Due to heart failure/cardiomyopathy	5 (0.6%)	18 (0.6%)
Prior to or after transplantation	4 (0.5%)	13 (0.4%)
Other	13 (1.6%)	18 (0.6%)

**Findings**		
Normal/atheroma	273 (34.1%)	602 (19.3%)
1-vessel disease	285 (35.6%)	1066 (34.2%)
2-vessel disease	119 (14.9%)	638 (20.5%)
3-vessel disease	70 (8.8%)	552 (17.7%)
Left main + 1 VD	10 (1.3%)	30 (1.0%)
Left main + 2 VD	12 (1.5%)	65 (2.1%)
Left main + 3 VD	19 (2.4%)	138 (4.4%)
Left main	8 (1.0%)	13 (0.4%)

**Primary decision**		
PCI ad hoc	441 (55.1%)	2003 (64.3%)
No intervention	124 (15.5%)	338 (10.9%)
Continued medical treatment	96 (12.0%)	291 (9.3%)
Continued examination	95 (11.9%)	293 (9.4%)
Coronary artery bypass graft grafting	17 (2.1%)	111 (3.6%)
Elective PCI	4 (0.5%)	25 (0.8%)
Valve surgery	3 (0.4%)	6 (0.2%)
Other surgical intervention	17 (2.1%)	28 (0.9%)

*Abbreviations:* STEMI, ST-Elevation Myocardial Infarction; NSTEMI, Non-ST-Elevation Myocardial Infarction; VD, Vessel Disease; Left main indicates left main coronary artery; PCI, percutaneous coronary intervention.

### Association between female sex and coronary intervention

In the logistic regression analysis, women were less likely to undergo coronary angiography both in the crude model and after adjustments for age, calendar year, comorbidities, and presenting rhythm (OR 0.77, 95% CI 0.66–0.90) (Table 4). Furthermore, female sex was associated with lower odds of performance of both PCI and CABG (Table 4). When adjusting for coronary angiographic findings, there were no significant differences in the performance of PCI between women and men (OR 0.97, 95% CI 0.80–1.18).

**Table 4:** Odds ratios of coronary angiography and reperfusion therapy in women compared to men after resuscitated out-of-hospital cardiac arrest.

**Outcome**	**Women**	**Men**	Adjusted OR*	Adjusted OR**
Coronary angiography	800	3114	0.56 (0.49–0.63)	0.77 (0.66–0.90)
PCI	441	2003	0.56 (0.48–0.65)	0.76 (0.64–0.90)
CABG	31	197	0.58 (0.43–0.78)	0.60 (0.42–0.83)

*Abbreviations:* OR, Odds ratio; PCI, percutaneous coronary intervention; CABG, coronary artery bypass grafting.

* Adjusted for age, calendar year and comorbidities.

** Adjusted for age, calendar year, comorbidities and first rhythm.

### Patients presenting with non-shockable rhythm and no ST-segment elevation

Of 3914 patients undergoing coronary angiography, 9.7% (*n* = 381) presented with non-shockable rhythm and no ST-segment elevation (34.9% women [*n* = 133]). As expected, this subgroup of patients had more comorbidities (Supplementary Table 1). Moreover, they were less likely to have a witnessed cardiac arrest, suffer the cardiac arrest in a public location and were less frequently discharged alive.

The most common indication for coronary angiography in this subgroup was “cardiac arrest without STEMI”, representing 61.7% of the performed angiographies in women and 62.9% in men (Table 5). Most of these revealed normal coronary arteries or mild atheroma, reported in 69.2% of women and 46.0% of men. Single‑vessel disease was found in 13.5% of women and 20.2% of men, while two‑vessel disease occurred in 5.3% and 12.1%, respectively. Three‑vessel disease was identified in 5.3% of women and 13.7% of men. Left main stem involvement was uncommon overall but tended to be more frequent in men.

**Table 5:** Coronary angiography indications, findings and primary decision in 381 OHCA patients presenting with non-shockable rhythm and no ST-segment elevation.

	**Women**	**Men**
Total	133 (34.9%)	248 (65.1%)

**Indication**		
Cardiac arrest w/o STEMI	82 (61.7%)	156 (62.9%)
Cardiac arrest, undefined	30 (22.6%)	59 (23.8%)
NSTEMI/unstable angina pectoris	4 (3.0%)	9 (3.6%)
Due to heart failure/cardiomyopathy	1 (0.8%)	3 (1.2%)
Due to arrhythmia	2 (1.5%)	1 (0.4%)
Prior to or after transplantation	3 (2.3%)	7 (2.8%)
Other	9 (6.8%)	11 (4.4%)

**Findings**		
Normal/atheroma	92 (69.2%)	114 (46.0%)
1-vessel disease	18 (13.5%)	50 (20.2%)
2-vessel disease	7 (5.3%)	30 (12.1%)
3-vessel disease	7 (5.3%)	34 (13.7%)
Left main + 1 VD	0 (0.0%)	3 (1.2%)
Left main + 2 VD	3 (2.3%)	4 (1.6%)
Left main + 3 VD	4 (3.0%)	8 (3.2%)
Left main	2 (1.5%)	2 (0.8%)

**Primary decision**		
No intervention	47 (35.3%)	76 (30.6%)
PCI ad hoc	28 (21.1%)	71 (28.6%)
Continued medical treatment	20 (15.0%)	41 (16.5%)
Continued examination	27 (20.3%)	45 (18.1%)
Coronary artery bypass grafting	0 (0.0%)	4 (1.6%)
Other surgical intervention	10 (7.5%)	9 (3.6%)

*Abbreviations:* STEMI, ST-Elevation Myocardial Infarction; NSTEMI, Non-ST-Elevation Myocardial Infarction; VD, Vessel Disease; Left main indicates left main coronary artery; PCI, percutaneous coronary intervention.

Reperfusion therapy were relatively similar between sexes with ad hoc PCI performed in 21% of women and 29% of men in this group (Table 5). No intervention was chosen in roughly one third of cases, and continued medical treatment and further diagnostic evaluation were selected at similar rates between sexes.

## Discussion

The main result of this study was that women are significantly less likely than men to undergo coronary angiography following resuscitated OHCA compared to men, regardless of age, pre-existing comorbidities, or presenting rhythm at the time of the arrest.

Our results are consistent with previous studies conducted in Europe, the United States, and South Korea,^20^, ^21^, ^22^, ^23^, ^24^ all of which report that women have significantly lower odds of receiving coronary angiography and reperfusion therapy after OHCA with ROSC, even after adjustments for potential confounders and resuscitation characteristics. Furthermore, in line with earlier findings, we observed no significant sex differences in the likelihood of undergoing PCI once coronary angiography was performed.^20^, ^21^, ^25^

There are several possible explanations for the observed difference in coronary angiography use between women and men in our study. Current post-resuscitation guidelines recommend considering coronary angiography in patients with ROSC after OHCA without ST-elevation on the ECG if there is a high probability of an acute coronary occlusion.^26^ Given that ischemic heart disease is more prevalent in men, clinicians may be more prone to suspect a coronary aetiology in male patients, influencing decision-making in the emergency setting. Moreover, prior studies have reported that women with OHCA are less likely to present with STEMI, a key determinant of urgent coronary angiography.^20^, ^23^ In our study, the proportion of STEMI did not differ between sexes, however this observation was limited to patients who actually underwent coronary angiography. Sex differences may still be present earlier in the clinical pathway, contributing to the lower overall use of invasive coronary evaluation in women. Although we adjusted for known ischemic heart disease, this may not fully capture undiagnosed or acute coronary syndromes occurring at the time of the arrest. Furthermore, we observed that men had more extensive CAD, whereas women had a higher prevalence of normal angiograms. This finding aligns with previous studies on cardiac arrest patients undergoing coronary angiography,^11^, ^25^ and may in part contribute to sex differences in diagnostic and treatment trajectories.

Unlike our findings, a previous Swedish study observed no sex differences in the utilization of coronary angiography following OHCA.^11^ However, that study was limited to patients presenting with a shockable initial rhythm, whereas our cohort included all initial rhythms, which possibly explains this discrepancy. The persistent sex difference in the use of coronary angiography, despite adjustments for age, comorbidities, and presenting rhythm, observed in this study raises concerns about potential under-treatment of women. There is evidence supporting the fact that women are less likely to receive guideline-recommended therapies following OHCA.^27^, ^28^ However, it remains unclear whether the observed sex difference in the present study reflects unmeasured actual differences in clinical presentation or unmeasured factors influencing clinical decision-making.

Although ischemic heart disease is the most common cause of sudden cardiac arrest,^29^, ^30^ differences in the underlying aetiology of the OHCA may have contributed to our results. For example, women in our cohort were more likely to have prevalent chronic obstructive pulmonary disease, suggesting a higher likelihood of a respiratory cause of the cardiac arrest. In addition, we observed that asphyxia was more commonly reported as the cause of the arrest among women. In such cases, the decision to forgo coronary angiography may be clinically appropriate, reflecting a lower suspicion of an acute coronary artery occlusion. However, the true aetiology of the cardiac arrest is often difficult to determine in the acute setting, and decisions must frequently be made with limited information.

This was the first study to report sex-specific coronary angiographic findings and management in OHCA patients presenting with non-shockable rhythm and no ST-segment elevation. These patients accounted for only one in ten of coronary angiographies performed in this study. Women comprised a substantial proportion of this group however (34.9%), consistent with the well-established association between female sex and non-shockable rhythms. Although this subgroup frequently demonstrated normal coronary arteries, it is noteworthy that within this clinically low-suspicion group, significant CAD was present in approximately one third of women and more than half of men. These findings align with previous work reporting that among the few non-shockable, no ST-segment elevation OHCA patients undergoing coronary angiography, almost 30% have an acute culprit lesion.^13^, ^15^

OHCA patients presenting with non-shockable rhythms and no ST-segment elevation constitute a large group and clinically important group, accounting for nearly 45% of all OHCA patients admitted alive to hospital.^13^ Despite absence of demonstrated benefit from routine early angiography in randomized trials of OHCA without ST-segment elevation, many of these studies included no or very small numbers of patients with non-shockable rhythms, and most did not report coronary angiographic findings stratified by presenting rhythm.^31^, ^32^, ^33^, ^34^ In contrast, the EMERGE study,^35^ in which most patients presented with non-shockable rhythms, reported that although nearly half of patients had normal coronary angiograms, approximately 25% underwent PCI, findings that closely align with our results. Similarly, a re-analysis of the PEARL study showed that the small subset of patients with non-shockable rhythm had a prevalence of culprit lesions comparable to those presenting with shockable rhythms.^36^ Taken together, these findings highlight the need of improved risk stratification tools to identify which patients with non‑shockable OHCA and no ST‑segment elevation may benefit from invasive coronary evaluation, as a non‑shockable rhythm alone does not exclude the presence of significant CAD. Ongoing randomized trials, including the DISCO study (ClinicalTrials.gov ID: NCT02309151), are expected to provide important evidence to guide the management of this heterogeneous patient group.^37^

A major strength of this study was the use of comprehensive nationwide health care registries, ensuring complete follow-up and minimizing selection bias. The large sample size enhanced the statistical power, while the use of validated healthcare registries with low rates of missing data reduced the risk of misclassification. Confounding was addressed through multivariable regression models.

There are limitations to be mentioned. First, as an observational study, we cannot rule out residual confounding or selection bias, despite adjustments for known covariates. Second, we lacked reliable data on resuscitations variables such as the duration of CPR, time to ROSC, and CPR quality, all of which could influence both survival and clinical decision-making regarding coronary angiography. Third, we did not have access to ECG findings for patients who did not undergo coronary angiography, neither did we have echocardiographic data. This is a significant limitation, as ECG and echocardiographic findings are central to the decision to perform immediate coronary angiography and potential PCI. Fourth, we lacked detailed information on the in-hospital clinical course following ROSC. It is therefore possible that women were in a poorer clinical state than men post-resuscitation, which may have influenced the decision regarding invasive procedures such as coronary angiography. However, the absence of a notable difference in 1-day survival between sexes questions the assumption that women presented in a significantly worse clinical condition than men.

To conclude, in this nationwide study of patients with OHCA admitted alive to hospital, we found that women were less likely than men to undergo coronary angiography, even after adjusting for age, comorbidities, and presenting rhythm. We found that women and men had similar indications for coronary angiography in terms of ECG findings, although women had more normal coronary angiograms than men. Among patients presenting with non-shockable rhythm and no ST-segment elevation undergoing coronary angiography, we found that significant CAD remained common and that approximately 1 in 4 of these patients required PCI. Our results highlight the need for increased awareness of potential sex-based disparities in post-resuscitation care and emphasize the need for improved risk stratification to identify which non-shockable non-ST-segment elevation patients may benefit from invasive coronary evaluation.

## Declaration of generative AI and AI-assisted technologies in the manuscript preparation process

During the preparation of this work the author(s) used Microsoft Copilot to improve the quality of the English in the article. After using this tool/service, the author(s) reviewed and edited the content as needed and take(s) full responsibility for the content of the published article.

## CRediT authorship contribution statement

**Charlotte Miedel:** Writing – original draft, Visualization, Project administration, Methodology, Investigation, Formal analysis, Data curation, Conceptualization. **Lis Abazi:** Writing – review & editing, Validation, Resources, Investigation, Data curation. **Sune Forsberg:** Writing – review & editing, Validation, Resources, Investigation, Data curation. **Martin Jonsson:** Writing – review & editing, Validation, Resources, Methodology, Investigation, Formal analysis, Data curation, Conceptualization. **Gabriel Riva:** Writing – review & editing, Validation, Supervision, Resources, Project administration, Methodology, Investigation, Funding acquisition, Data curation, Conceptualization.

## Declaration of competing interest

The authors declare that they have no known competing financial interests or personal relationships that could have appeared to influence the work reported in this paper.

## Appendix A Supplementary material

Supplementary Data 1 Subgroup analysis and ICD-codes used in the study.
