# Supplementary material for: Coronary angiography and reperfusion therapy following out-of-hospital cardiac arrest in women
Source: Resusc Plus. 2026 Aug 24;31:101462. doi: 10.1016/j.resplu.2026.101462 (PMC13572081; doi:10.1016/j.resplu.2026.101462)
Supplement: Supplementary file 1 [file mmc1.docx]

**Supplementary material**

**Supplementary Table 1. Baseline Characteristics Among 381 Resuscitated OHCA Patients With Non-shockable Rhythm and no ST-segment Elevation Undergoing Coronary Angiography**

| **Characteristic** | **Women** | **Men** |
| --- | --- | --- |
| Total | 133 (34.9%) | 248 (65.1%) |
| Age, median [IQR] | 65 [57-72] | 67 [58-72] |
| Hypertension | 58 (43.6%) | 115 (46.4%) |
| Diabetes mellitus | 29 (21.8%) | 64 (25.8%) |
| Ischemic heart disease | 25 (18.8%) | 82 (33.1%) |
| Heart failure | 22 (16.5%) | 45 (18.1%) |
| Chronic kidney disease | 3 (2.3%) | 12 (4.8%) |
| Chronic obstructive pulmonary disease | 20 (15.0%) | 20 (8.1%) |
| Smoking status |  |  |
| Never smoked | 17 (12.8%) | 26 (10.5%) |
| Ex smoker >1 month | 25 (18.8%) | 36 (14.5%) |
| Current smoker | 20 (15.0%) | 40 (16.1%) |
| Unknown | 71 (53.4%) | 146 (58.9%) |
| Previous PCI | 11 (8.3%) | 35 (14.1%) |
| Previous CABG | 4 (3.0%) | 21 (8.5%) |
| **Resuscitation characteristics** |  |  |
| Witnessed arrest | 105 (78.9%) | 199 (80.2%) |
| Bystander CPR | 70 (52.6%) | 146 (58.9%) |
| Location of the arrest |  |  |
| At home | 98 (73.7%) | 153 (61.7%) |
| Public | 17 (12.8%) | 75 (30.2%) |
| Other | 18 (13.5%) | 20 (8.1%) |
| Cardiac arrest etiology |  |  |
| medical | 123 (92.5%) | 226 (91.1%) |
| trauma | 1 (0.8%) | 6 (2.4%) |
| intox | 3 (2.3%) | 5 (2.0%) |
| drowning | 0 (0.0%) | 1 (0.4%) |
| asphyxia | 2 (1.5%) | 5 (2.0%) |
| other | 0 (0.0%) | 1 (0.4%) |
| **Survival** |  |  |
| Discharged alive | 30 (22.6%) | 55 (22.2%) |

Abbreviations: IQR, interquartile range; CPR, cardiopulmonary resuscitation; PCI, percutaneous coronary intervention; CABG, coronary artery bypass grafting.

**Supplementary Table 2. ICD-10 codes for Confounders.**

| **Comorbidity** | **ICD-10** |
| --- | --- |
| Hypertension | I10 |
| Diabetes Mellitus | E10, E11 |
| Congestive heart failure | I50 |
| Ischemic heart disease | I21, I25 |
| Chronic obstructive pulmonary disease | J44 |
| Chronic kidney disease | N18, N19 |
